# Lysine Methylation and Histone Modifications during Cold Stress of Insects: Freeze-Tolerant *Eurosta solidaginis* and Freeze-Avoiding *Epiblema scudderiana*

**DOI:** 10.3390/insects15070498

**Published:** 2024-07-04

**Authors:** Zhijun Yu, Tingwei Pei, Han Wang, Chunyuan Wang, Jingze Liu, Kenneth B. Storey

**Affiliations:** 1 Hebei Key Laboratory of Animal Physiology, Biochemistry and Molecular Biology, Hebei Collaborative Innovation Center for Eco-Environment, Hebei Research Center of the Basic Discipline of Cell Biology, Ministry of Education Key Laboratory of Molecular and Cellular Biology, College of Life Sciences, Hebei Normal University, Shijiazhuang 050024, China; 2 Institute of Biochemistry, Carleton University, 1125 Colonel By Drive, Ottawa, ON K1S 5B6, Canada

**Keywords:** histone acetylation, histone methylation, epigenetics, diapause, cold stress

## Abstract

**Simple Summary:**

Insects, like other animals, must face severe winter low temperature conditions in northern areas. Some insects even freeze entirely and wait out the winter until spring comes. Epigenetic modifications have been suggested to play a vital role in regulating the cold responses of insects. However, the dynamics of the enzymes involved in histone lysine methylation, histone H3 acetylation and methylation in response to cold winters in the freeze-tolerant goldenrod gall fly larvae *Eurosta solidaginis* and the freeze-avoiding gall moth caterpillars *Epiblema scudderiana* remains unclear. This paper investigates the modifications of key nuclear proteins that act as gene regulators and mainly focuses on proteins—called histones—that act to depress metabolism. The results indicate that histones are modified in *E. solidaginis* and *Ep. scudderiana* during winter. Significant reductions were observed in most of the targets of histone methylation/acetylation for decreasing temperatures of *Ep. scudderiana* larvae, whereas selected histone methylation/acetylation targets were conversely elevated in *E. solidaginis*. These epigenetic changes are shown to be an important element in winter survival.

**Abstract:**

Overwintering survival by insects, whether of the freeze-tolerant or freeze-avoiding types, is typically associated with a strong suppression of metabolic rate (e.g., entry into diapause) that involves the differential expression of many genes with regulation at the transcriptional, translational or post-translational levels. Epigenetic modifications have been suggested to play a vital role in regulating cold responses of insects. However, knowledge of the roles of epigenetic mechanisms in modulating gene expression for winter survival of the larvae of two goldenrod gall formers, the freeze-tolerant dipteran *Eurosta solidaginis* and the freeze-avoiding lepidopteran *Epiblema scudderiana*, remain unknown. The current study evaluates the role of cold-induced lysine methylation and histone modifications, with enzymes of lysine methylation (SETD8, SETD7, SUV39H1, SMYD2 and ASH2L), as well as relative levels of histone H3 acetylation (H3K9ac, H3K18ac, H3K27ac, H3K56ac) and methylation (H3K4me1, H3K9me3, H3K36me2) examined in two insects. Significant (*p* < 0.05) reductions were observed in most of the targets of histone methylation/acetylation for decreasing temperatures of *Ep. scudderiana* larvae, whereas selected histone methylation/acetylation targets were conversely elevated (*p* < 0.05) in *E. solidaginis*, particularly under conditions of 5 °C for 4 h. Histone H3 expression was found to be variable without statistical differences in larval goldenrod gall moths and gall flies. These results provide basic information on the patterns of epigenetic regulation involved in insect cold hardiness.

## 1. Introduction

Epigenetics mainly refers to the heritable and stable changes in gene expression that occur through alterations in the chromosome rather than in the DNA sequence, and epigenetic modifications such as DNA methylation, histone post-translational modifications, chromatin remodeling and noncoding RNAs play important roles in regulating the gene activity states of organisms under endogenous or exogenous stresses, mediating the signals arising from developmental, metabolic and environmental cues [1]. DNA methylation and histone modifications are the best-known epigenetic regulatory mechanisms [2]. DNA methylation is achieved by groups of DNA methyltransferases (DNMTs), widely recognized as mediating transcriptional regulation in response to environmental signals and can also mediate organized and programmed response to predictable and specific environmental signals [3]. The functions of DNA methylation have been widely recognized in plants, animals and microorganisms [4]. However, DNA methylation in insects is found to be present at considerably lower levels than in plants and mammals and is exclusively confined to gene bodies [5], with the function relating to plasticity and regulation of the phenotype of insects [6].

Histone modifications mainly refer to the modifications on the histone tails of chromatin proteins that influence chromatin structure, which includes the acetylation and methylation of lysines (K) and arginines (R) [7]. Some other histone modifications, such as the phosphorylation of serine, threonine and tyrosine, ubiquitylation and sumoylation of lysines, as well as ribosylation are also observed [8]. They affect the transcriptional state and expression level of the genes that they guard [9]. Two groups of enzymes including histone acetyltransferases (HATs) and histone deacetylases (HDACs) are included in the transfer of acetyl groups to and from histones [10]. However, histone methylation has proven to be more complex, with different degrees of methylation (mono-, di-, or trimethylation) on lysine residues and arginine residues, which display vastly different functions [11]. In insects, histone modifications have been found play significant roles in the regulation of development and reproduction [12].

Recently, the definition of epigenetics has also been expanded to include noncoding RNAs (ncRNAs) as well as modifications to mRNA (e.g., methylation of adenosine) or methylation on the arginine moieties of proteins [13]. Additionally, chronic cold exposure was found to improve insulin sensitivity, with the involvement of DNA methylation and histone deacetylation [14]. Intriguing intersections between different epigenetic mechanisms are becoming better recognized [15], and research on the role of epigenetic modifications in insects will help to understand the ecological and evolutionary strategies for survival [16].

Winter is a challenging time for insect species living at high latitudes or altitudes and yet many species survive these challenges, some being capable of enduring temperatures as low as –70 °C [17]. Although some species winter as eggs, many others overwinter as larvae, pupae or adults and employ adaptive strategies involving behavioral, physiological and/or biochemical mechanisms to achieve cold hardiness. Many species enter a diapause (quiescent) state and generally use one of two strategies: freeze tolerance (enduring freezing of extracellular body fluids) or freeze avoidance (employing deep supercooling of body fluids) with well-known survival strategies including upregulation of antifreeze or ice-nucleating proteins and accumulation of high levels of cryoprotectants (e.g., glycerol, sorbitol and others) [18,19]. Epigenetic modifications have also been suggested to play a role in regulating the cold responses of insects [20]. Previous study found that small RNAs and histone modifications are involved in the pupal diapause of the flesh fly *Sarcophaga bullata* [21,22]. Histone methylation, alternative splicing and small RNAs were found to be involved in the diapause of *Chymomyza costata* [23]. Random epigenetic alterations were also found during the transgenerational changes in the cold hardiness of *Aedes albopictus* eggs [24].

The freeze-tolerant goldenrod gall fly larvae *Eurosta solidaginis* (Fitch, 1855) and the freeze-avoiding gall moth caterpillars *Epiblema scudderiana* (Clemens, 1860) are two well-studied model insects whose larvae overwinter in galls on the stems of goldenrod plants (*Solidago canadensis*), and their biochemical and physiological changes against winter low temperature have been extensively explored [25,26,27]. In addition, previous studies found upregulation of select DNA methyltransferase (DNMT) enzymes with concurrent decreases in the DNMT activity of *Ep. scudderiana* under subzero temperature exposure but no change in the activity of the Ten-Eleven Translocation (TET) demethylation enzyme activities [28], whereas the epigenetic modifications of the enzymes involved in histone lysine methylation, histone H3 acetylation and methylation in response to cold winter remains unknown. Hence, in the present study, both species were acclimated under short (4 h) and long (≥1 week) exposures to decreasing environmental temperatures (15 °C, 5 °C and −15 °C). The protein expression levels of five lysine methyltransferases were determined and the post-translational acetylation and methylation modifications on histone H3 were assessed. The above results will expand our knowledge on the potential roles of epigenetic regulation in insect overwintering strategies.

## 2. Materials and Methods

### 2.1. Insect Collection and Cold Stress

The galls of two insect species, the larvae of the freeze-tolerant goldenrod gall fly (*E. solidaginis*) occupying ball galls and caterpillars of the goldenrod gall moth (*Ep. Scudderiana*) which occupy the elliptical galls, were collected from fields around Ottawa (Latitude: 45.41, Longitude: −75.67), Ontario, Canada in mid-autumn (18–20 October) in 2006–2007. Insects in their galls were acclimated in an incubator at 15 °C for 3 weeks before sampling. Control larvae were randomly selected from this group, the galls being rapidly sliced-open and the larvae extracted and flash frozen in liquid nitrogen. Then, the incubation temperature was set at 5 °C, with incubation time lasting for 4 h or 3 weeks, and samples were collected as above. Subsequently, the incubation temperature was reduced to −15 °C and maintained for 4 h or 1 week, with sampling as descried above. All collected samples were placed at −80 °C until use.

### 2.2. Total Protein Isolation

After cold acclimation, 8–10 frozen larval individuals were quickly weighed (~0.5 g) and ground into powder using a mortar and pestle under liquid nitrogen. The powder was transferred into plastic tubes, followed by the quick addition (1:2 *w*:*v*) of homogenizing buffer (20 mM Hepes pH 7.5, 200 mM NaCl, 0.1 mM EDTA, 10 mM NaF, 1 mM Na_3_VO_4_, 10 mM β-glycerophosphate). Protease inhibitors (including 5.0 μL/mL of phenylmethylsulfonyl fluoride (PMSF, 200 mM) and 10.0 μL/mL of Sigma Protease Inhibitor) were also added. Then, the samples were immediately homogenized at 10,000 rpm for 15 s under normal model and centrifuged at 12,000× *g* at 4 °C for 15 min. The clear supernatant below a floating fat layer was transferred to new tubes and the protein concentrations were determined via the Bradford assay using Biorad reagent (Catalogue #: 500-0006), with bovine serum albumin used as standard. A final concentration of 8 μg/mL was prepared by adding the calculated volumes of homogenizing buffer. Aliquots of the standardized samples were then mixed 1:1 *v*:*v* with 2× SDS denaturing buffer (100 mM Tris-base, 4% *w*:*v* SDS, 20% *v*:*v* glycerol, 0.2% *w*:*v* bromophenol blue, 10% *v*:*v* 2-mercaptoethanol) and boiled for 10 min to denature proteins. Samples were then stored at −40 °C until use.

### 2.3. Western Immunoblotting

The protein levels of five histone methyltransferases (HMTs) (SETD8, SMYD2, SUV39H1, SETD8 and ASH2L) were assessed via immunoblotting, with four replicates carried out within each treatment. Samples of the soluble protein extracts (5–10 μL depending on the protein being analyzed) were loaded onto 10–15% SDS-polyacrylamide gels and electrophoresis was carried out for 85–130 min at 180V at room temperature using a BioRad Mini-Protean 3 System. To assess histone H3 methyl modifications, samples (10–15 μL) were loaded onto 15% Tris-Tricine gels and electrophoresed for 30 min at 60V, followed by 2.5 h at 160 V at 4 °C. For each sample, the same volume was added and the gel percentages and run times were kept consistent. On each gel, aliquots of 5 μL of pre-stained protein molecular weight ladder (Froggabio, Toronto, ON, Canada; Cat. # PM005-0500) were added.

After electrophoresis, the transfer of the proteins was carried out using BioRad Mini-Protean Transfer cells (BioRad, Mississauga, ON, Canada), and 0.45 μm Polyvinylidene fluoride (PVDF) membranes (Millipore, Billerica, MA, USA, Cat. #: IPVH00010) submerged in ~800 mL transfer buffer (20 L buffer consists of 60.6 g Tris-base, 288 g glycine, 4 L methanol and 16 L of ddH_2_O) were used, with the conditions set at 80–160 mA for 45–90 min. Subsequently, the PVDF membranes were washed in 0.5 × 10 mM tris(hydroxymethyl)aminomethane-HCl, 120 mM NaCl and 0.1% Tween 20, pH 7.5 (TBST buffer) for 5 min and then blocked with 0.5–12% milk for 10–30 min, followed by washing again for 3×5 min in 0.5×TBST. Then, the PVDF membrane was incubated overnight at 4 °C with primary antibody at a dilution of 1:1000 *v*:*v* ratio of antibody to TBST.

The antibodies used were as follows: ASH2L (#5019), SETD8 (#2996), SETD7 (#2813), SMYD2 (#9734), SUV39H1 (#8729), H3K9ac (#9649), H3K18ac (#13998), H3K27ac (#8173) and H3K56ac (#4243), all from Cell Signaling Technology (Danvers, MA, USA), as well as H3K4me1 (#ab8895) and H3K9me3 (ab8898) from Abcam (Cambridge, MA, USA) and H3K36me2 (#39256) from Active Motif (Carlsbad, CA, USA).

Following overnight incubation with the primary antibodies, all PVDF membranes were washed 3 times for 5 min each in 0.5×TBST to remove any nonspecific primary antibody. Subsequently, they were incubated at room temperature in diluted (1:5000 or 1:8000 *v*:*v*) HRP-conjugated anti-rabbit IgG secondary antibody (Bioshop, Burlington, ON, Canada #APA007P) in 0.5×TBST for 30 min. Then, three washes (5 min each with 0.5×TBST) were carried out on the membranes. A mixture of 600 μL Luminol and H_2_O_2_ (1:1 *v*:*v*) were used to incubate the membranes for 2–10 min, which were imaged in a Chemi-Genius Bio-Imaging System (Syngene, Frederick, MD, USA) using Gene Tools software 4.3.7 (Syngene, Cambridge, UK). After imaging, the membranes were stained with Coomassie blue (0.25% *w*:*v* Coomassie brilliant blue, 7.5% *v*:*v* acetic acid, 50% *v*:*v* methanol), with the protein bands re-imaged as above. For calculations, the band intensities on PVDF membranes were standardized against the total intensity of a group of Coomassie-stained protein bands found in all lanes that showed constant expression across all lanes, which were well-separated from the target band of interest.

### 2.4. Statistical Analysis

The data were tested for normality using the Shapiro–Wilk test, and comparisons among each group were carried out using one-way analysis of variance (ANOVA) and a Dunnett’s post-hoc test (*p* < 0.05) using GraphPad Prism software (version 8.03.3, San Diego, CA, USA). The histograms were constructed using Sigmaplot 12.0 software (Systat Software Inc., San Jose, CA, USA).

## 3. Results

### 3.1. Marked Differences in the Expression of Lysine Methyltransferases in Ep. scudderiana and E. solidaginis in Response to Low Temperature

**Figure 1:**
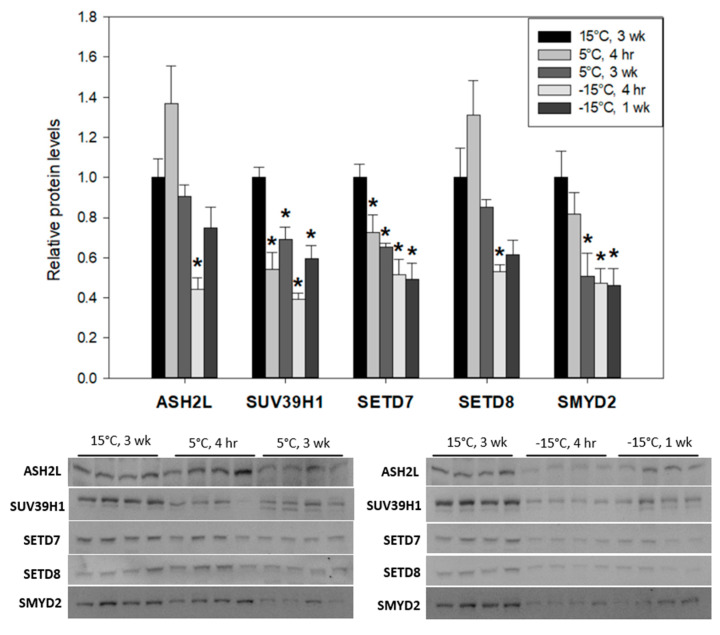
Relative expression levels of five lysine methyltransferases, ASH2L, SUV39H1, SETD7, SETD8 and SMYD2 in freeze-avoidant *Epiblema scudderiana* larvae after cold exposure at 5 °C for 4 h or 3 weeks or after freezing exposure at −15 °C for 4 h or 1 week. Histograms represent mean ± SEM; n = 4 from different insect samples. Band intensities on PVDF membranes were standardized against the total intensity of a group of Coomassie-stained protein bands found in all lanes that showed constant expression across all lanes, and the *y*-axis shows the relative abundance of the protein/post-translational modification of interest in each treatment. Data were analyzed via one-way ANOVA with post-hoc Dunnett’s tests. Treatments denoted with an asterisk (*) are significantly (*p* < 0.05) different from 15 °C, 3-week controls.

**Figure 2:**
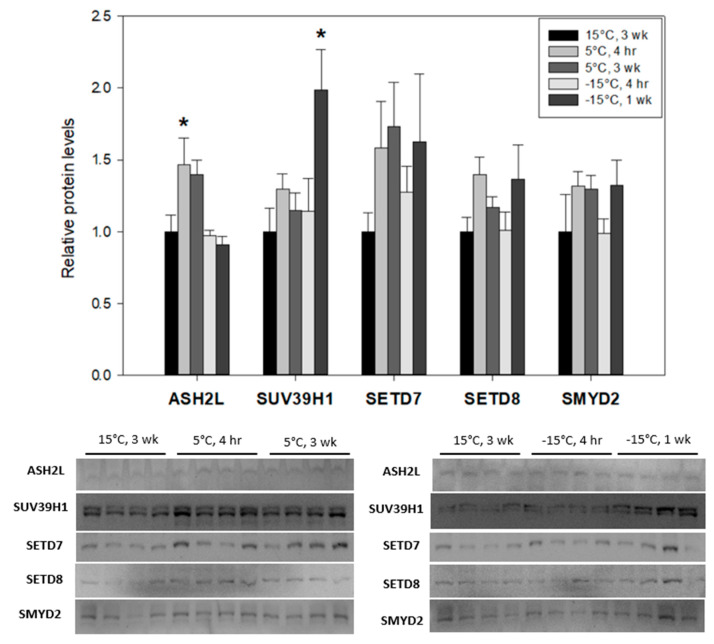
Relative expression levels of five lysine methyltransferases, ASH2L, SUV39H1, SETD7, SETD8 and SMYD2 in freeze-tolerant *Eurosta solidaginis* larvae after cold exposure at 5 °C for 4 h or 3 weeks or after freezing exposure at −15 °C for 4 h or 1 week. Treatments denoted with an asterisk (*) are significantly (*p* < 0.05) different from 15 °C, 3-week controls. Other information as in Figure 1.

Dynamic changes in the relative protein expression levels of ASH2L, SUV39H1, SETD7, SETD8 and SMYD2 were identified in larval *Ep. scudderiana* and *E. solidaginis* when comparing controls (held at 15 °C for 3 weeks) with cold-exposed (5 °C for 4 h or 3 weeks) and subzero-exposed larvae (−15 °C for 4 h or 1 week). Among gall moth larvae (*Ep. scudderiana)* that utilize freeze-avoiding strategies, all five proteins studied displayed downregulation as the temperature declined (Figure 1) from 15 °C to −15 °C. On the other hand, the freeze-tolerant larvae of gall flies were largely unresponsive to cold/freeze stress (Figure 2), with only ASH2L and SUV39H1 significantly upregulated under selected conditions. ASH2L, a core component of eukaryotic H3K4 methylating complexes, displayed a significant decrease to 44 ± 5% of controls (F_(4, 15)_ = 9.61, df = 15, *p* = 0.0092) in *Ep. scudderiana* larvae under subzero stress (−15 °C, 4 h), whereas the orthologous ASH2L protein in *E. solidaginis* was significantly elevated 1.5 ± 0.2-fold (F_(4, 15)_ = 5.61, df = 15, *p* = 0.031) following 4 h at 5 °C. Similar disparities were observed with SUV39H1. *Epiblema* SUV39H1 was highly responsive to decreasing larval temperatures and was significantly (F_(4, 15)_ = 13.93, df = 15, *p*_(15°C 3wk vs. 5°C 4h)_ = 0.003; *p*_(15°C 3wk vs. 5°C 3wk)_ = 0.0093; *p*_(15°C 3wk vs. −15°C 1wk)_ = 0.0010) depressed across all tested cold treatments, being depleted by 39 ± 3% of controls under −15 °C, 4 h conditions (F_(4, 15)_ = 13.93, df = 15, *p* < 0.0001). *Eurosta* SUV39H1, which produced a double band, was markedly elevated 2.0 ± 0.3-fold (F_(4, 15)_ = 4.06, df = 15, *p* = 0.009) during the deep freeze (−15 °C, 1 week), relative to controls.

SETD7 and SMYD2 were found to be decreased (SETD7: F_(4, 15)_ = 8.55, df = 15, *p*_(15°C 3wk_ vs. _5°C 4h)_ = 0.0473; *p*_(15°C 3wk vs. 5°C 3wk)_ = 0.0113; *p*_(15°C 3wk vs. −15°C 4h)_ = 0.0007; *p*_(15°C 3wk vs. −15°C 1wk)_ = 0.0005; SMYD2: F_(4, 15)_ = 5.47, df = 15, *p*_(15°C 3wk vs. 5°C 4h)_ = 0.5647; *p*_(15°C 3wk vs. 5°C 3wk)_ = 0.0153; *p*_(15°C 3wk vs. −15°C 4h)_ = 0.0095; *p*_(15°C 3wk vs. −15°C 1wk)_ = 0.0081) across increasingly more severe cold stresses, reaching their lowest (SETD7: 49 ± 8%, *p* = 0.0005; SMYD2: 46 ± 9%, *p* = 0.0081) after 1 week at −15°C (Figure 1), while SETD7 (F_(4, 15)_ = 0.94, df = 15, *p*_(15°C 3wk vs. 5°C 4h)_ = 0.4996; *p*_(15°C 3wk vs. 5°C 3wk)_ = 0.3096; *p*_(15°C 3wk vs. −15°C 4h)_ = 0.9179; *p*_(15°C 3wk vs. −15°C 1wk)_ = 0.4401) and SMYD2 (F_(4, 15)_ = 1.217, df = 15, *p*_(15°C 3wk vs. 5°C 4h)_ = 0.4468; *p*_(15°C 3wk vs. 5°C 3wk)_ = 0.5054; *p*_(15°C 3wk vs. −15°C 4h)_ > 0.9999; *p*_(15°C 3wk vs. −15°C 1wk)_ = 0.4389) expression in the freeze-tolerant gall fly larvae was unaffected following cold or frozen experimental exposures (Figure 2). SETD8, a H4K20-specific methyltransferase of vertebrate chromatin, was significantly (F_(4, 15)_ = 8.16, df = 15, *p* = 0.028) depressed in the −15 °C, 4 h conditions in *Epiblema* larvae (Figure 1) but not in *Eurosta* larvae (F_(4, 15)_ = 1.73, df = 15, *p* > 0.9999) (Figure 2).

### 3.2. The Trends of Histone Methylation Correlate with Methyltransferase Expression and Suggest Distinctions between the Responses of Freeze-Tolerant E. solidaginis and Freeze-Avoiding Ep. scudderiana to Low Temperature

**Figure 3:**
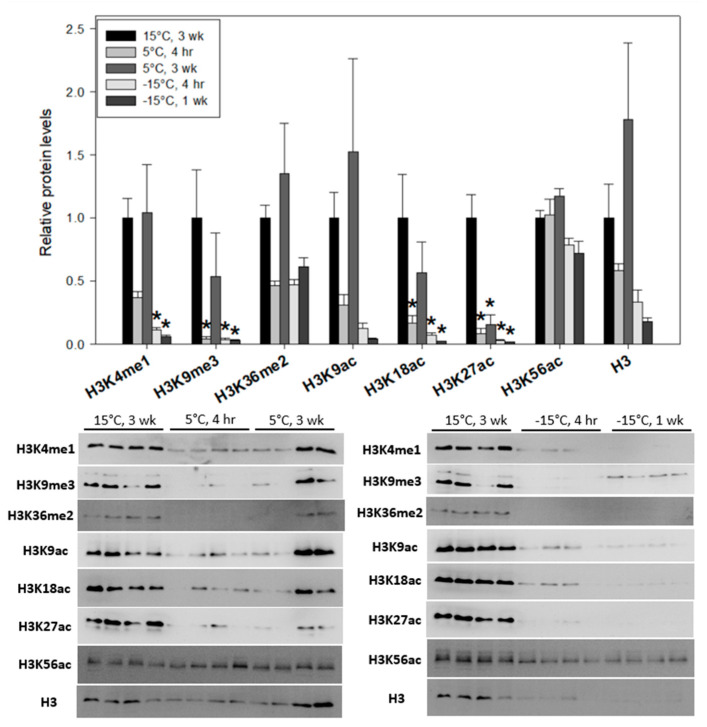
Relative abundance of histone H3 and notable H3 modifications (H3K4me1, H3K9me3, H3K36me2, H3K9ac, H3K18ac, H3K27ac, H3K56ac) in *Epiblema scudderiana* after cold (5 °C) or freezing (−15 °C) exposure. Treatments denoted with an asterisk (*) are significantly (*p* < 0.05) different from 15 °C, 3-week controls. Other information as in Figure 1.

Three prominent histone methyl-lysine modifications (H3K4me1, H3K9me3 and H3K36me2) were observed in *Ep. scudderiana* and *E. solidaginis* larvae exposed to decreasing experimental temperatures (5 °C for 4 h or 3 weeks and −15 °C for 4 h or 1 week). H3K4me1 and H3K9me3 displayed similar dynamic changes in larval *Ep. scudderiana* under increasing cold exposure (Figure 3). When stressed at 5 °C for 4 h, the methylation levels of H3K4me1 and H3K9me3 were strongly suppressed downwards to 35 ± 5% and 10 ± 2% as compared to 15 °C controls, respectively, but rose upwards to near control levels when 5 °C was maintained for 3 weeks. Interestingly, when *Epiblema* larvae were transferred to −15 °C, the methylation levels of H3K4me1 (F_(4, 15)_ = 7.32, df = 15, *p* = 0.01) and H3K9me3 (F_(4, 15)_ = 5.04, df = 15, *p* = 0.01) decreased sharply and remained at roughly only 10% that of control larvae. However, the abundance of H3K36me2 was statistically unchanged across all tested cold treatments (Figure 3).

**Figure 4:**
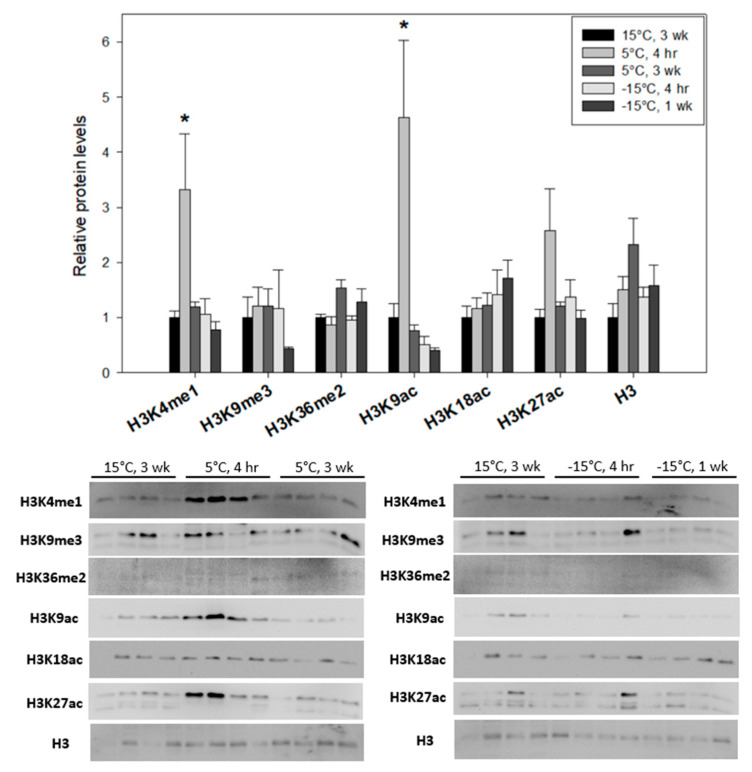
Relative abundance of histone H3 and notable H3 modifications (H3K4me1, H3K9me3, H3K36me2, H3K9ac, H3K18ac, H3K27ac) in *Eurosta solidaginis* after cold (5 °C) or freezing (−15 °C) exposures. Treatments denoted with an asterisk (*) are significantly (*p* < 0.05) different from 15 °C, 3-week controls. Other information as in Figure 1.

Conversely, the levels of H3K4me1 in larval *E. solidaginis* were significantly (F_(4, 15)_ = 4.92, df = 15, *p* = 0.01) elevated (3.4 ± 1.0-fold) at 5 °C for 4 h as compared to 15 °C controls but returned to normal levels across all other cold treatments (Figure 4). H3K36me2, a histone modification associated with repressed gene transcription, trended upwards following prolonged 3-week exposures to 5 °C (Figure 4). There were no statistical differences in H3K9me3 prevalence among the tested experimental conditions (F_(4, 15)_ = 0.65, df = 15, *p* = 0.638).

### 3.3. Opposing Levels of Histone Acetylation in Larval E. solidaginis and Ep. scudderiana in Response to Low-Temperature Exposure

The relative expression levels of histone H3 and acetylated H3 modifications (H3K9ac, H3K18ac, H3K27ac and H3K56ac) in larval *Ep. scudderiana and E. solidaginis* were measured after cold stress at 5 °C for 4 h, 5 °C for 3 weeks, −15 °C for 4 h or −15 °C for 1 week. Histone H3 expression was variable in larval goldenrod gall moths (Figure 3) and gall flies (Figure 4); however, in neither species did the changes reach statistical significance (*Eurosta*: F_(4, 15)_ = 2.27, df = 15, *p* = 0.11; *Epiblema*: F_(4, 15)_ = 4.53, df = 15, *p* = 0.208). The non-significant changes in the histone H3 levels in *Epiblema* were primarily due to the large biological variations of the 5 °C, 3-week larval samples. However, the levels of H3K9ac, H3K18ac and H3K27ac all decreased strongly after (1) 4 h at 5 °C, (2) 4 h at −15 °C and (3) 1 week at −15 °C as compared with controls (Figure 3). The prevalence of these three modifications all reached their lowest levels following −15 °C, 1-week incubations, which were all less than 5% of normal. Interestingly, H3K27ac was the only modification of these three that did not return to control levels during prolonged (3-week) 5 °C conditions. The H3K9ac changes among treatments were not statistically significant due to the large biological variation of 5 °C, 3-week larval samples. H3K56ac, which was only detected in *Epiblema* larvae via immunoblotting, was significantly unresponsive to any cold treatment (Figure 3).

In *E. solidaginis*, H3K9ac and H3K27ac were both conversely elevated in larvae following initial (4 h) exposures to 5 °C (4.6 ± 1.0-fold; F_(4, 15)_ = 7.78, df = 15, *p* = 0.04 and 2.6 ± 0.8-fold; F_(4, 15)_ = 2.99, df = 15, *p* = 0.035, respectively) before subsequent reductions back to normal levels across 5 °C 3-week, −15 °C 4 h and −15 °C 1-week treatments (Figure 4). No significant changes were observed in H3K18ac levels across any treatments (F_(4, 15)_ = 0.91, df = 15, *p* = 0.48) (Figure 4).

## 4. Discussion

The winter survival of insects, using either a freeze-tolerant or freeze-avoiding strategy, is usually associated with significant metabolic rate depression caused not just by low temperatures but often also by entry into a diapause state [29,30]. Multiple changes in gene expression can occur, and such changes can be regulated at the transcriptional, translational and post-translational levels [31,32]. The current study investigated the effects of cold exposure on lysine methylation and histone modifications in two goldenrod gall formers, *Ep. scudderiana* (freeze avoiding) and *E. solidaginis* (freeze tolerant), experiencing cold stress. These epigenetic modifications can provide reversible regulation of metabolism for long-term survival under winter cold conditions when the larvae of both species are in a diapause state.

It is widely known that SETD8, SMYD2, SUV39H1, SETD7 and ASH2L control transcriptional activity via their methyltransferase actions on the N- and C-terminal tails of histone proteins in nucleosomes [32]. In *Drosophila* cells, depletion of SETD8 resulted in the accumulation of DNA damage and an ATR-dependent cell cycle arrest [33]. SMYD2 is a histone 3 methyltransferase that can also methylate other proteins such as p53, Rb and HSP90 [34]. SUV39H1 has been found highly associated with H3K9me3, a chromatin marker that can suppress nearby gene transcription [35]. SETD7 is characterized by unique enzymatic activity and protein domain architecture, which features histone H3K4 mono-methyltransferase associated with transcriptional activation [36]. In the current study, the levels of most of these enzymes were unchanged in *Ep. scudderiana* larvae during initial (4 h) exposures to 5 °C; however, all five methyltransferases showed significant downregulation at one or both sampling times when larvae were moved to −15 °C (Figure 1). Gall moth SUV39H1 and SETD7 orthologs were particularly cold responsive and were significantly (*p* < 0.05) depressed across all cold treatment time points. The decreased SUV39H1 and SETD7 expression correlates strongly with their putative histone targets, H3K9me3 [37] and H3K4me1 [38], respectively (Figure 3). In gall moth freeze avoidance, the downregulation of the lysine methyltransferases SETD7 and SUV39H1 likely contributes to the depletion of H3K4me1 and H3K9me3 on chromatin, exemplifying the importance of gene regulatory controls in support of the transition to a dormant hypometabolic state.

By contrast, *E. solidaginis* larvae displayed a distinct epigenetic signature in response to decreasing experimental temperatures, which is likely explained by the differing cellular phenotypes of the freeze-tolerant and freeze-avoiding strategies. None of the five tested methylase enzymes had decreased protein levels in any cold treatment conditions (Figure 2), and this corresponds with an overall maintenance of the histone H3 methyl-lysine modifications H3K4me1, H3K9me3 and H3K36me2 (Figure 4). Interestingly, ASH2L, a core component of evolutionarily conserved SET1 methyltransferase, has been found to possess an intrinsic methyltransferase activity for proper H3K4 tri-methylation in *Drosophila* [36,39,40]. It exhibited a lone significant (*p* < 0.05) upregulation in protein expression upon the initial cold (5 °C) exposure of *E. solidaginis*, which is associated with the cold-mediated induction of H3K4me1 levels in gall fly larvae. These results suggest that activation of SET1 complexes through upregulated ASH2L expression may drive elevated H3K4me1, a permissive histone modification that helps facilitate the necessary transcriptional activation of cold-tolerance genes at the onset of cold exposure. The strong increase in another permissive modification, H3K9ac, which is similarly elevated in 5 °C, 4 h gall fly larvae (Figure 4), re-enforces this idea. Changes in H3K9ac were also found in *Anopheles gambiae* upon *Plasmodium falciparum* infection [41]. In contrast, no difference in H3K9ac abundance was found in *Drosophila melanogaster* across diapausing and persistently reproductive ovaries [42].

Strong reductions in global gene transcription are speculated to contribute to the metabolic suppression of insect diapause [43]. In the Colorado potato beetle, *Leptinotarsa decemlineata*, downregulation of gene expression has been found between the diapause initiation and maintenance phases [44]. Frozen (−15 °C, 1 week) *E. solidaginis* larvae in diapause exhibited an increase in SUV39H1 protein expression (Figure 2). SUV39H1 activity is highly associated with transcriptionally repressed heterochromatin [31]. Therefore, upregulation of SUV39H1 in 1-week frozen gall flies may contribute to transcriptional gene silencing in support of the freeze-tolerant strategies of *E. solidaginis*.

Despite statistically unchanged levels of total histone H3 in both the gall moth (Figure 3) and gall fly (Figure 4) larvae, the relative abundance of specific H3 acetyl-lysine modifications showed key differences across the various cold treatments. H3K18ac and H3K27ac were highly cold responsive in *Ep. scudderiana* larvae but not in *E. solidaginis* larvae. H3K18ac and H3K27ac are enriched near transcriptional start sites (TSS) of active gene promoters across eukaryotic life [45]. H3K18ac levels were found to be significantly higher on onset and lower with time extension in diapause-type pupae than in the non-diapause cotton bollworm *Helicoverpa armigera* [46]. H3K27ac modification has also been observed in *Drosophila* [47]. The strong depletion of the permissive modifications H3K18ac and H3K27ac in freeze-avoiding *Epiblema* larvae at −15 °C is indicative of transcriptionally suppressed heterochromatin in favor of insect diapause. Finally, H3K56ac was only detected in *Ep. scudderiana* (Figure 3) and not in *E. solidaginis* (Figure 4), which may be due to the differing prevalence of these specific modifications in orthologous chromatin.

## 5. Conclusions

The present study found significant reductions in most of the targets of histone methylation/acetylation for decreasing temperatures of *Ep. scudderiana* larvae, whereas selected histone methylation/acetylation targets were conversely elevated in *E. solidaginis*, particularly under 5 °C, 4 h conditions. The above results provide evidence in favor of the epigenetic transcriptional regulation of insect diapause and outlines key differences in the epigenetic signatures of freeze-tolerant and freeze-avoiding overwintering strategies.

## Data Availability

The data presented in this study are available on request from the corresponding authors.
